# Bridging the Gap in Emergency Medicine in Pakistan

**DOI:** 10.5811/westjem.2019.10.44502

**Published:** 2020-01-27

**Authors:** Syed G. Saleem, Kaniz F. Haider, Saima Salman, Lubna Samad, Zayed Yasin, Megan Rybarczyk

**Affiliations:** * The Indus Hospital, Department of Emergency Medicine, Karachi, Pakistan; † Indus Health Network, Center for Essential Surgical and Acute Care, Karachi, Pakistan; ‡ The Indus Hospital, Department of Pediatric Surgery, Karachi, Pakistan; § Medtigo, North Adams, Massachusetts; ¶ Brigham and Women’s Hospital, Department of Emergency Medicine, Division of Global Emergency Care and Humanitarian Studies, Boston, Massachusetts

To the Editor:

Pakistan has an increasing need for a strong emergency care system as emergency conditions – acute cardiovascular disease, road injuries, and stroke – form the top 10 leading causes of death.^1^ However, until 2019 the country had only seven officially-recognized emergency medicine (EM) residency programs, on average five years long, leading to an insufficient number of EM specialists, and a large gap in quality emergency care provision. This, in addition to the high turnover rate of the existing emergency doctors, results in a gap that will take approximately 30 years to bridge.^2^ Hence, in the interim, a training module is needed, comprising of a well-developed, shorter EM curriculum to efficiently train the current emergency department (ED) workforce: predominantly medical officers with no formal EM training.

Thus, the year-long Certification Program in Emergency Medicine (CPEM) was developed by EM specialists from The Indus Hospital (TIH), Karachi, Pakistan, and Brigham & Women’s Hospital – a teaching affiliate of Harvard Medical School, Boston, USA - and launched at TIH in July 2018.

The objectives of this program are to ensure that participants become familiar with fundamental concepts in EM, understand ED processes and patient flows, seek formal specialisation in EM, represent and advocate for EM over various platforms, and, overall provide better patient care.

The teaching site is a 300-bed tertiary care facility, with a 25-bed ED, six-bed intensive care unit, six-bed coronary care unit, and five operating rooms, serving a low-income catchment population and approximately 300,000 patients monthly. The CPEM is aimed towards training EM physicians in proper emergency care, improving ED employee retention, and fostering support for EM throughout Pakistan. The program curriculum is based on the College of Physicians & Surgeons Pakistan (CPSP), the American College of Emergency Physicians (ACEP) and African Federation of Emergency Medicine (AFEM) guidelines, with input from EM specialists experienced in providing and managing EM training in both first-world and lower-and-middle-income country (LMIC) contexts. The curriculum is divided into 12 month-long topic blocks, each based around a specialty or organ system (eg, cardiovascular, trauma, psychiatry) (Table 1).

CPEM offers both didactic and practical learning to full-time physicians from TIH and from five other institutes across Karachi, with instruction and mentorship from local and international faculty. Participants are divided into two groups: CPEM-Clinical (CPEM-C), comprised of doctors from the teaching site, and CPEM-Didactic (CPEM-D) (ie, doctors from the other hospitals). Participants are assessed through regular examinations and formative and summative evaluations. Other competencies, such as participants’ attitude, professionalism, documentation, cognitive processes, etc., are also assessed for quality improvement purposes using guidelines developed by the Accreditation Council for Graduate Medical Education (ACGME) in the USA. Special innovations within CPEM include point-of-care ultrasound practice, flipped classroom sessions, practical workshops (eg, intubation, splinting and reduction, laceration repair), weekly case-based discussions over a messaging application, and use of low-cost improvised models to hone procedural skills (eg, thoracostomy, lateral canthotomy, incision and drainage, central venous catheter placement). CPEM-C participants receive clinical mentorship in the ED from the Visiting Faculty. Additionally, participants are also given exposure to various types of imaging and technology (e.g., computed tomography, ultrasound, and radiograph; magnetic resonance imaging is not available at the teaching site).

Another innovative aspect is the proactive role of the participants. Their feedback is used in program decision-making and curriculum revision, and the institutional diversity they bring allows for a healthy exchange of ideas, practices and policies, all of which contribute towards a dynamic and efficient learning experience. This will eventually lead to improved quality of care, and stronger inter-ED synergy in the future.

Throughout the first academic year, from July 2018 to June 2019, participants had received nearly 300 hours of instruction, and covered over 70 simulated cases, with CPEM-C trainees additionally logging several hundred cases and supervised procedures. In its first year, CPEM graduated 25 out of 32 originally enrolled physicians, with about 20 participants certain about seeking additional training in EM. In its new academic year, CPEM has 29 enrollees, from eight different hospitals, with some excelling graduates from the first batch returning to assist as instructors.

Ultimately, as this model embodies a modular, flexible learning approach, with a concentration on adaptation vs adoption, it has the potential to be replicated in other settings with a high burden of emergency conditions and rudimentary emergency care systems. It is hoped that the CPEM model can be expanded to other hospitals and will foster increased inter-ED collaboration, and continued interest in EM will contribute towards significantly advancing the quality and accessibility of emergency care in Pakistan. Especially in LMICs, where EM is still emerging, it will take decades to achieve a sufficient capacity of formally trained providers. However, the CPEM model can serve as a feasible mechanism in bridging this gap and helping to improve the overall state of emergency care in low-resource settings.

For more information on CPEM, please visit: http://www.cpem.com.pk/.

## Figures and Tables

**Table 1:** Certification Program in Emergency Medicine Block Overview.

Block	Topic	Key skills and procedures
1	Cardiovascular	Advanced Cardiac Life Support (light), echocardiogram, electrocardiogram (ECG), pericardiocentesis, central venous catheter placement, ultrasound-guided intravenous line placement, ankle-brachial indices, pulsus paradoxus
2	Pulmonary	Intubation, mechanical ventilation, non-invasive positive pressure ventilation, arterial blood gas, chest tube/needle decompression, thoracentesis, lung ultrasound
3	Trauma	Advanced Trauma Life Support (light), FAST/e-FAST
4	Orthopedics, Immunology/Rheumatology, Dermatology	Arthrocentesis, laceration repair, wound care, incision and drainage, procedural sedation, nerve blocks, splinting, joint radiograph interpretation
5	Renal, Genitourinary, Gynecology	Foley placement, renal/bladder/pelvic ultrasound, lab interpretation (electrolytes)
6	Pediatrics	Pediatric Advanced Life Support (light), intraosseous line placement, pediatric lumbar puncture, pediatric intravenous access, pediatric ultrasound, pediatric radiograph interpretation
7	Obstetrics/Gastrointestinal	ALSO (light), emergency delivery, obstetrical ultrasound, nasogastric tube placement, paracentesis, abdominal ultrasound
8	Neurology	NIHSS, lumbar puncture
9	Ophthalmology and HEENT	Slit lamp exam, foreign body removal, peritonsillar abscess drainage, nasal packing, lateral canthotomy, dental block, eye ultrasound
10	Hematology, Oncology, Endocrinology	Lab interpretation (hematology and coagulation studies, endocrinology studies)
11	Psychiatry and Toxicology	Chemical and physical restraints, ECG interpretation
12	Infectious Diseases	Antibiotic use, review of ultrasound and procedures

*ECG*, electrocardiogram; *FAST*, focused assessment with sonography in trauma; *e-FAST*, extended focused assessment with sonography in trauma; *ALSO*, Advanced Life Support in Obstetrics; *NIHSS*, National Institutes of Health Stroke Scale; *HEENT*, head, eyes, ears, nose, and throat.
